# Knowledge of health risks, safety practices, acute pesticide poisoning, and associated factors among farmers in rural irrigation areas of northeastern Ethiopia

**DOI:** 10.3389/fpubh.2024.1474487

**Published:** 2024-11-21

**Authors:** Awoke Keleb, Ayechew Ademas, Masresha Abebe, Gete Berihun, Belay Desye, Anmut Endalkachew Bezie

**Affiliations:** ^1^Department of Environmental Health, College of Medicine and Health Sciences, Wollo University, Dessie, Ethiopia; ^2^Department of Occupational Health and Safety, College of Medicine and Health Sciences, Wollo University, Dessie, Ethiopia

**Keywords:** associated determinant factors, knowledge, safety practices, pesticides, acute pesticide poisoning acute pesticide poisoning, Ethiopia, farmers, health risks

## Abstract

**Background:**

Pesticide exposure is a major public health issue among farmers affecting make it their health, economic stability, and agricultural productivity. This study aimed to investigate the magnitude and determinants of farmers’ knowledge of health risks, safety practices, and acute pesticide poisoning in the South Wollo Zone, Ethiopia.

**Methods:**

A community-based cross-sectional study was conducted in the South Wollo Zone’s irrigation areas from July to August 2022. Using a multistage sampling technique, five out of ten irrigation districts were randomly selected, and three kebeles (the smallest administrative unit) from each district (15 total) were chosen based on intensive irrigation activities. A proportional sample size was allocated across the selected kebeles according to the number of farmers. Trained data collectors conducted face-to-face interviews using a pretested structured questionnaire. Crude and adjusted odds ratios with 95% confidence intervals at a *p*-value of 0.05 identified statistically significant factors.

**Result:**

The study found that 55.2% of farmers had below mean knowledge of health risks, 63.7% practiced below mean safety practices, and 47.9% experienced acute pesticide poisoning. Significant factors for below mean knowledge included no formal education (AOR = 2.32; 95% CI: 1.27–4.23) and lack of pesticide training (AOR = 2.07; 95% CI: 1.33–3.21). Below mean safety practices were associated with age > 47 years (AOR = 2.07; 95% CI: 1.06–4.04), below mean retailer actions (AOR = 1.97; 95% CI: 1.34–2.89), and below mean attitude (AOR = 1.79; 95% CI: 1.23–2.62). Acute pesticide poisoning was significantly associated with over 10 years of exposure (AOR = 4.34; 95% CI: 2.55–7.39), below mean knowledge (AOR = 2.17; 95% CI: 1.23–2.62), lack of training (AOR = 3.73; 95% CI: 2.33–5.98), and below mean safety practices (AOR = 4.40; 95% CI: 2.86–6.78).

**Conclusion:**

Farmers’ knowledge of health risks and safety practices was not satisfactory. Low educational status, lack of training, and minimal government involvement were associated with below mean knowledge. Below mean knowledge and below mean safety practices, low education, lack of training, and minimal government involvement were associated with acute pesticide poisoning. Young farmers, limited retailer involvement, and below mean attitudes contribute to unsafe practices.

## Introduction

Pesticides are used to prevent or control pests in areas such as agriculture, animal husbandry, and public health (1, 2). Pesticides are now an essential component of modern farming as a result of rapid population growth, and the rise of commercial agriculture, particularly in irrigation areas to boost crop productivity (1). However, the extensive and careless use of pesticides worldwide endangers human health and the environment, particularly in cases of severe pesticide poisoning (3, 4).

Acute pesticide poisoning (APP) is any disease or health effect brought on by a suspected or verified pesticide exposure within 48 h (5). It is a serious occupational and public health issue that affects millions of people worldwide, especially farmers who are exposed to pesticides through occupational activities, and is mostly prevalent in developing nations (1, 4, 6). APP can cause various symptoms such as nausea, vomiting, headache, dizziness, seizures, coma, and even death. The long-term impacts of APP may also affect the reproductive system, nervous system, and mental health (7, 8).

It is highly probable that farmers are exposed to pesticides through contact with pesticide residues on treated crops, unsafe handling, improper pesticide storage (9–11) dietary residues, and contamination of drinking water (12–14). They are also exposed due to unsafe application of pesticides to household animals or below mean disposal techniques (15), neglecting to maintain spraying equipment, and not using protective equipment properly (6, 12, 16, 17). These risk s could be made worse by farmers’ negative attitudes and below mean perceptions of the risks of pesticide exposure, as well as their ignorance about health concerns and usage (18–20).

Moreover, misuse or pesticide use without safety in developing nations includes using pesticides that the local government has banned, leaking from excessive spraying (21), failure to use personal protective device for self-defense (16), improper pesticide storage, mixing and applying pesticides with bare hands, and, in the worst case scenario, reusing pesticide containers for food and drinking water (2, 17, 22). Furthermore, farmers’ ability to protect themselves from risks associated with pesticides is severely hampered by incorrect perceptions regarding pesticide use, which increase the risk of APP and its complications (23, 24).

Acute pesticide poisoning is often underdiagnosed and underreported, making it difficult to estimate the true burden of the problem and to implement effective prevention strategies. However, according to a WHO and United Nations Environment Programme report, 3 million individuals worldwide are exposed to pesticides, which is attributed to 200,000 annual deaths (7). In Africa, there has been a rise in acute poisoning from exposure to toxic pesticides, as documented by several nations. Kenya recorded 1,479 cases and 579 fatalities from pesticide poisoning in 2012, while Uganda recorded roughly 87 fatalities from the same cause in 2013 (25).

According to data from poison control centers, pesticide poisoning accounts for 3.6% of adults and 3.4% of pediatrics deaths, with a total proportion of 3.3% from all types of accidental poisoning (26). The health effects of pesticide use in developing nations are mostly caused by illiteracy, lack of training in pesticide use (27), lack of information about risks and misuse, improper handling of pesticides, improper handling of pesticide containers, poor pesticide selection, and quality, ignorance, and poor safety practices (24, 27, 28).

The previous studies indicated that the prevalence of pesticide-related morbidity and mortality is higher among rural farmers than urban farmers due to illiteracy, lack of access to safety equipment, and safety instructions in local language on pesticide containers, making them difficult to understand (29–31). The agricultural sector is the backbone for over 80% of the Ethiopian population to maintain food security and other amenities (2, 20, 32, 33).

The increased demand for productivity and rural irrigation has increased dramatically during the past 10 years, involving tens of thousands of farmers highly dependent on extensive pesticide utilization in Ethiopia, including the South Wollo zone. However, scientific evidence on the level of knowledge of health risks, safety practices, and associated health effects among rural farmers in irrigation areas (2, 10, 34) remains limited in Ethiopia.

These issues, in turn, prompt the following three immediate research questions: First, how well-informed are pesticide users about the potential health risks? Secondly, what safety practices have farmers taken to safeguard themselves against recognized health risks? And finally, how many pesticide-user farmers experienced acute pesticide poisoning? Hence, this study aims to determine the level of farmers’ knowledge of health risk, safety practices, acute pesticide poisoning, and associated factors among farmers in irrigation areas of South Wollo Zone, north-eastern Ethiopia. The evaluation of knowledge, attitude, and safety practices towards pesticide use and acute pesticide poisoning due to occupational exposure among farmers is very essential to designing cost-effective public health interventions and strategies.

## Methods

### Study design, study area, and source population

**Figure 1:**
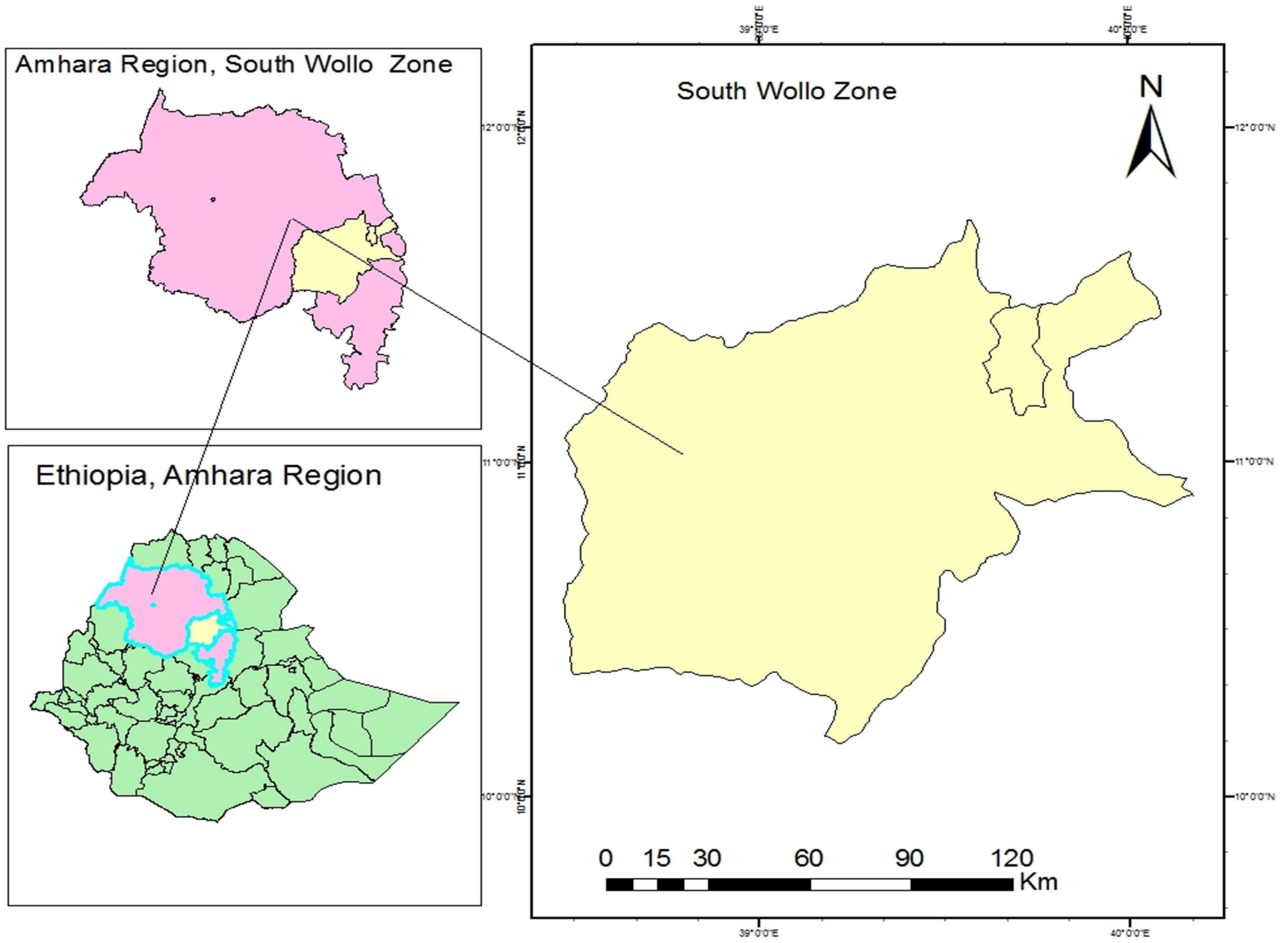
Map of study area.

A community-based cross-sectional study was conducted in rural irrigation areas of the South Wollo Zone from July to August 2022. South Wollo is one of ten zones in the Amhara region of Ethiopia. South Wollo is bounded on the south by North Shewa and the Oromia special zone; on the west by East Gojjam; on the north by North Wollo; on the northeast by Afar Region; and on the southeast by the Oromia Zone and the Argobba special woreda (Figure 1).

The South Wollo Zone has a total of 24 districts with 2,518,862 people in its catchment area, of which 301,638 (11.98%) live in urban areas and 2,217.224 (88.02%) in rural areas. Its area is 17,067.45 square km, and its population density is 147.58 (35). Agriculture is the main industry in the South Wollo Zone. A wide variety of agricultural systems, from large-scale commercial farms to small-scale subsistence farming, are practiced in the zone.

A range of crops are extensively irrigated using pesticides because the rainfall in the area is erratic and insufficient. Pesticides are widely used in irrigation agriculture to increase productivity in the South Wollo zone, despite their detrimental effects on the environment and human health. Cereals, including teff, wheat, and barley, as well as fruits, vegetables, and other cash crops, are the primary crops grown using irrigation. For this study, the source populations included farmers in 10 districts with irrigation activities out of 20 districts in South Wollo zone, whereas the study population was farmers in eight randomly selected districts from eighteen districts with intensive irrigation activities and pesticide use carried out in South Wollo zone, Northeastern Ethiopia.

### Sample size and sampling procedure

A total of 626 study participants were determined by Epi Info software version 7.0, using a single population proportion equation by considering local assumptions (36).


$$n=\frac{{\left(z_{a/2}\right)}^{2}*p\left(1-p\right)}{d^{2}}$$

Where: *n*: is the adequate sample size required, *Z_α/2_* is the standard normal variable at (1-α) % confidence level (α is 0.05 with 95%CI, Z_α/2_ = 1.96), *p* is an estimate of the proportion (54.4%) of farmers with good knowledge of pesticide control methods (6) and *d* is the margin of error 5%. Based on these assumptions, 379 farmers were determined.

Considering design effect 1.5, *n* = 380 * 1.5 = 570. Finally, to reduce errors arising from the probability of non-compliance, 10% of the sample size contingency was added so that the final sample size was determined to be 627.

A multistage sampling technique was used to select five districts randomly from 10 irrigation districts in the South Wollo Zone, followed by three kebeles (the smallest administrative unit in Ethiopia) from each district (a total of 15 kebeles were taken) selected randomly from all kebeles with intensive irrigation activities. A proportional sample size allocation was made across each of the selected kebeles based on the number of farmers in each kebele. This method ensures that districts with more irrigation farmers have a proportionally larger representation in the sample, improving the accuracy and reliability of the study results. To assure randomization, houses and farmers were selected using a computer-generated pseudo-random number method. First, we randomly selected houses inside each kebele. The head of the household or another adult member over the age of 18 who was in charge of farming activities was eligible to participate in the study.

### Study variables

The dependent variables were farmers’ knowledge of health risk (below mean or good), safety practice (below mean or good), and acute pesticide poisoning (yes or no). Independent variables were organized into three parts: Part I contained socio-economic and demographic variables; Part II consisted of health and safety-related variables, including duration of exposure, knowledge of pesticide use, the action of retailers, the action of the government, and training on proper pesticide use; and Part III consisted of symptoms of acute pesticide poisoning.

#### Below mean knowledge of health risk

Each knowledge measurement indicator was given a value of 1 for the positive action or correct response and 0 for the negative action or incorrect response. The sum of these response values was calculated, and the mean score of the responses was used as a cut-off point to categorize them as below mean knowledge or above mean knowledge. Farmers’ reported less than the mean score of all knowledge measurement questions were considered to have below mean knowledge of health risks associated with pesticide use (17, 22).

#### Below mean attitude towards pesticide use

Farmers reported less than the mean scores of cumulative attitude measurement questions were considered to have below mean attitude towards pesticide use (17, 22).

#### Above mean action of retailers among farmers

Retailers who acted more than the mean score of the cumulative action of retailers’ measurement questions were considered to have above mean action in pesticide use (16).

#### Above mean action of government among farmers

A local government that acted more than the mean score of the cumulative action of government measurement questions was considered to have above mean action in pesticide use (16).

#### Acute pesticide poisoning

It was determined using self-reported main symptoms typically associated with pesticide use, including headache, ocular irritation, vision disturbance, airway irritation, dizziness, nausea/vomiting, diarrhea, skin lesion/irritation, abdominal cramp/pain, confusion/disturbance of memory, cough, and others. Based on the WHO standard case definition, a possible APP case is defined as two or more symptoms that occur within 48 h of pesticide use in the last twelve (13) months. An outcome variable was coded into two categories: “0” for “one’s report or no report of symptoms” and “1” for a possible APP case. Farmers who reported two or more symptoms within 48 h of pesticide use in the last 12 months were considered to have acute pesticide poisoning (5).

#### Model household

It is households who attended above 75% of training or 96 h of the 16 health packages and then certified as model households (observation of the certificate is mandatory), whereas a non-model household is a household head or caregiver who has taken basic training on the 16 health extension packages (37).

### Data collection tool and quality assurance

A structured questionnaire for this study was adapted from the WHO field survey of Exposure to Pesticides Standard Protocol and similar studies (15, 16, 38). The questionnaire was first prepared in English, translated to Amharic (the local language), and then translated back to English to ensure consistency. The reliability score (Cronbach *α* value) of the measurement question on knowledge of health risk, attitude towards pesticide use, and safety practice were 0.82, 0.75, and 0.87, respectively.

Six well-trained occupational health and safety professionals who had a bachelor’s degree and long experience in data collection were recruited as data collectors. Three days of extensive training were given to the data collectors and supervisors by the principal investigator before the start of the data collection process using a training manual. Training mainly focused on interviewing techniques and detailed discussion of each question, including those that require careful attention and observation. The training included lectures, mock interviews, and field practice.

To establish face validity and ensure study participants understood what the investigators intended to measure, a pre-test with 5% (30) of the total study sample was conducted in the nearby district of the study area. Data collectors performed the pre-test to familiarize themselves with the questionnaire and gain more experience with data collection. Pre-test results led to amendments to some items of the questionnaire.

For the purpose of ensuring data quality, regular supervision, monitoring, and random spot checks were conducted thoroughly by two environmental health experts with master’s degrees. Every day before entering the data, the supervisors and/or principal investigator reviewed the completed questionnaires to ensure that they were clear and complete. Every error was fixed the following day.

### Data management and analysis

Data were entered thoroughly using EpiData version 4.6 and exported to Statistical Package of Social Science (SPSS) version 25.0 for data cleaning and analysis. Quality control measures, including data cleaning using sorting, graphical exploration of distributions and cross-tabulations, and summary statistics, were performed. Descriptive statistics were used for categorical variables and mean ± SD (standard deviations) for continuous variables. Continuous variables were categorized using information from the literature, and categorical variables were re-categorized accordingly.

Binary logistic regression was used to analyze factors associated with knowledge of health risk, safety practices, and acute pesticide poisoning due to pesticide use among farmers. Three independent models were constructed for performing this regression analysis. The first, second, and third models were adjusted for the candidate variable to predict the extent of statistical association with knowledge of health risk, safety practices, and acute pesticide poisoning, respectively.

From the bivariable logistic analysis, variables with *p <* 0.25 were considered to select candidate variables for multivariable logistic analysis for knowledge of health risk (Model I), safety practice (Model II), and acute pesticide poisoning (Model III). Bivariable [crude odds ratio (COR)] and multivariable [adjusted odds ratio (AOR)] values were calculated using logistic regression analysis with a 95% confidence interval [CI].

From the multivariable logistic regression analysis, variables with a *p* value <0.05 were taken as statistically significant and independently associated with knowledge of health risk, safety practices, and acute pesticide poisoning. Multi-collinearity among independent variables was checked using standard error with cut-off point value 2 and variance inflation factor (VIF) less than 10. None of the variables were collinear based on the above checking criteria. Model fitness was also checked using the Hosmer-Lemeshow test, which had a *p*-value of 0.05 for each outcome variable.

## Results

### Socio-demographic variables of respondents

A total of 614 out of 626 households participated in the study with 97.9% response rate. Non-responses were due to refusals, interruptions, or households being closed after multiple visits. Of the respondents, 222 (36.2%) had ≥5 family members, and 326 (53.1%) were model households. The mean age of the participant was 32.31 ± 8.85 years, with 50.7% aged 28–37. The sample included 410 males (mean age 32.57 ± 9.26) and 204 females (mean age 31.78 ± 7.96). Nearly half (48.7%) were married, and 42.8% were illiterate. Principal component analysis categorized 205 households (33.4%) in the lowest quintile, 209 (34.0%) in the middle, and 200 (32.6%) in the highest quintile (Table 1).

**Table 1:** Socio-demographic characteristics of farmers in rural irrigation areas of South Wollo zone, Ethiopia, July–August, 2022.

Variable	Category	Frequency	Percentage (%)
Type of household	Non-model	288	46.9
	Model	326	53.1
Age of respondents’	18–27	184	30.0
	28–37	311	50.7
	38–47	57	9.3
	>47	62	10.1
Sex of the respondents’	Male	410	66.8
	Female	204	33.2
Marital status of the respondents’	Currently married	299	48.7
	Currently unmarried	315	51.3
Educational status of the household heads’	Unable to read and write	263	42.8
	Can read & write only	123	20.0
	Elementary education	89	14.5
	Secondary education	73	11.9
	College/university	66	10.7
Family size	>5 members	222	36.2
	≤5 members	392	63.8
Economic status	Lowest	205	33.4
	Medium	209	34.0
	Highest	200	32.6

### Health and safety-related factors

Fruits and vegetables were grown by more than half of the farmers, 320 (52.1%), followed by cereals and legumes, 176 (25.7%). Two hundred forty-eight (40.4%) and 140 (22.8%) farmers had exposure to pesticides for 4–10 and > 10 years, respectively. Nearly three-quarters of farmers, 450 (73.3%), had formal training on the proper use of pesticides. About half of pesticide retailers, 318 (51.8%), and 207 (33.7%) of local governments reported that they were not involved in providing information on how to use pesticides for farmers (Table 2).

**Table 2:** Health and safety related characteristics among farmers in rural irrigation areas of South Wollo zone, Ethiopia, July–August, 2022.

Variable	Category	Frequency	Percentage (%)
Types of crop grown	Cereals and legumes	176	28.7
	Fruits and vegetables	320	52.1
	Cash crops	118	19.2
Years of exposure	≤3 years	226	36.8
	4–10 years	248	40.4
	>10 years	140	22.8
Training about use of pesticides	No	450	73.3
	Yes	164	26.7
Action of retailor for farmers	Poor	318	51.8
	Good	296	48.2
Trust in the retailors information	Poor	186	30.3
	Good	428	69.7
Action of government for farmers	Poor	207	33.7
	Good	407	66.3
Trust in the government for farmers	Poor	213	34.7
	Good	401	65.3
Knowledge of health risk	Poor	339	55.2
	Good	275	44.8
Attitude towards pesticide use	Poor	332	54.1
	Good	282	45.9
Safety practice	Poor	391	63.7
	Good	223	36.3

### Knowledge of health risk measurement indicators

We calculated the mean score of 7.12 ± 2.61 out of a possible range of 0–14 for the cumulative knowledge score (actual range: 1–14). According to this study report, the majority of study participants were aware that using pesticides could have long-term effects on health, 355 (57.8%), short-term effects on health, 312 (50.8%), and potentially fatal intoxications, 80 (13.0%), as well as an adverse effect on water sources, 395 (64.3%), livestock, 369 (60.1%), and pollinating insects, 318 (51.8%).

Moreover, 320 (52.1%) and 236 (38.4%) of farmers knew pesticides could accumulate in fruits and vegetables and the soil, respectively. About 249 (40.6%) of participants recognized the skin as the main route of exposure for pesticides in the human body, whereas 382 (62.2%) and 351 (57.2%) reported the mouth and nose as possible routes of entry, respectively (Table 3).

**Table 3:** Knowledge of health risk associated with pesticide use among 614 farmers in rural irrigation areas of South Wollo zone, Ethiopia, July–August, 2022.

Assessment indicators (Items)	No (%)	Yes (%)
Pesticides causes health effects for applicators	259 (42.2)	355 (57.8)
Pesticides enter into the body through the skin	365 (59.4)	249 (40.6)
Pesticides enter into the body through the mouth	232 (37.8)	382 (62.2)
Pesticides enter into the body through the nose	263 (42.8)	351 (57.2)
Pesticides affect livestock	245 (39.9)	369 (60.1)
Pesticides affect pollinating insects (e.g., bees)	296 (48.2)	318 (51.8)
Pesticides affect water sources	219 (35.7)	395 (64.3)
Pesticides accumulate in fruits and vegetables	294 (47.9)	320 (52.1)
Pesticides induce only short-term effects	302 (49.2)	312 (50.8)
Pesticides accumulate in the soil	378 (61.6)	236 (38.4)
Pesticides that are banned or restricted for use	283 (46.1)	331 (53.9)
Pesticides cause potentially lethal intoxications	534 (87.0)	80 (13.0)
Pesticides should be handled only using PPE	174 (28.3)	440 (71.7)
Pesticides affects only children and elders	378 (61.6)	236 (38.4)
Overall knowledge using mean score and standard deviation	Poor	339 (55.2)
	Good	275 (44.8)

### Safety practice measurement indicators

We calculated the mean score and standard deviation of 4.55 ± 2.06 out of a possible range of 0–8, for cumulative safety practice (actual range: 0 to 8). More than two-thirds (417, or 67.9%) of farmers wore masks, gloves, long sleeves, and impermeable clothing when spraying pesticides. However, fewer than half of farmers carefully stored pesticides in safe containers after purchase. Less than half of the total study farmers, 279 (45.4%), used pesticides in excess of what was advised by local agricultural extensions or instruction manuals. However, only 361 farmers (58.8%) chose new pesticides that agricultural extension specialists recommended (Table 4).

**Table 4:** Safety practice associated with pesticide use among 614 farmers in rural irrigation areas of South Wollo zone, Ethiopia, July–August, 2022.

Assessment indicators (Items)	No (%)	Yes (%)
Wearing masks, gloves, and long-sleeved, and impermeable clothes when spraying pesticides	197 (32.1)	417 (67.9)
Changing clothes or showering immediately after spraying pesticides	440 (71.7)	174 (28.3)
Carefully storing pesticides in a safe place after a purchase	329 (53.6)	285 (46.4)
Never discarding the empty pesticide containers in the field after use	248 (40.4)	366 (59.6)
Never applying pesticides more than prescribed by agricultural extension or the instruction manual	279 (45.4)	335 (54.6)
Selecting new types of pesticides recommended by agricultural extension	361(58.8)	253 (41.2)
Low toxicity is the main reason for purchasing pesticides	468 (76.2)	146 (23.8)
Reading the instructions on the pesticide carefully before spraying	164 (26.7)	450 (73.3)
Overall safety practice using mean score and standard deviation	Poor	391 (63.7)
	Good	223 (36.3)

### Self-reported symptoms of acute pesticide poisoning

A total of 294 (47.9%) of farmers reported that they experienced at least two or more symptoms of acute pesticide poisoning within 48 h in the previous year after applying or handling pesticides, while 320 (52.1%)c of respondents did not attribute it to acute poisoning due to pesticide exposure. As shown in Table 4, nausea/vomiting, 248 (40.4%), cough, 240 (39.1%), airway irritation, 238 (38.8%), ocular irritation, 186 (30.3%), blurred vision, 169 (27.5%), shortness of breath, 159 (25.9%), diarrhea, 152 (24.8%), and skin lesions and irritation, 151 (24.6%), were the most commonly manifested symptoms of APP. Abdominal cramp 149 (24.3%), dizziness 122 (19.9%), and body tremor 118 (19.2%) were the least common symptoms reported by the farmers (Table 5).

**Table 5:** Prevalence of acute pesticide poisoning and symptoms distribution among 614 farmers in rural irrigation areas of South Wollo zone, Ethiopia, July–August, 2022.

Symptoms	No (%)	Yes (%)
Cough	374 (60.9)	240 (39.1)
Ocular irritation	428 (69.7)	186 (30.3)
Blurred Vision	445 (72.5)	169 (27.5)
Airway irritation	376 (61.2)	238 (38.8)
Shortness of breath	455 (74.1)	159 (25.9)
Nausea/vomiting	366 (59.6)	248 (40.4)
Diarrhoea	462 (75.2)	152 (24.8)
Skin lesion/irritation	463 (75.4)	151 (24.6)
Abdominal cramp/pain	465 (75.7)	149 (24.3)
Dizziness	492 (80.1)	122 (19.9)
Body tremor	490 (9.8)	118 (19.2)
Acute Pesticide Poisoning based on case possible case definition	No	320 (52.1)
	Yes	294 (47.9)

### Magnitude of knowledge, safety practice, and self-reported acute pesticide poisoning

**Figure 2:**
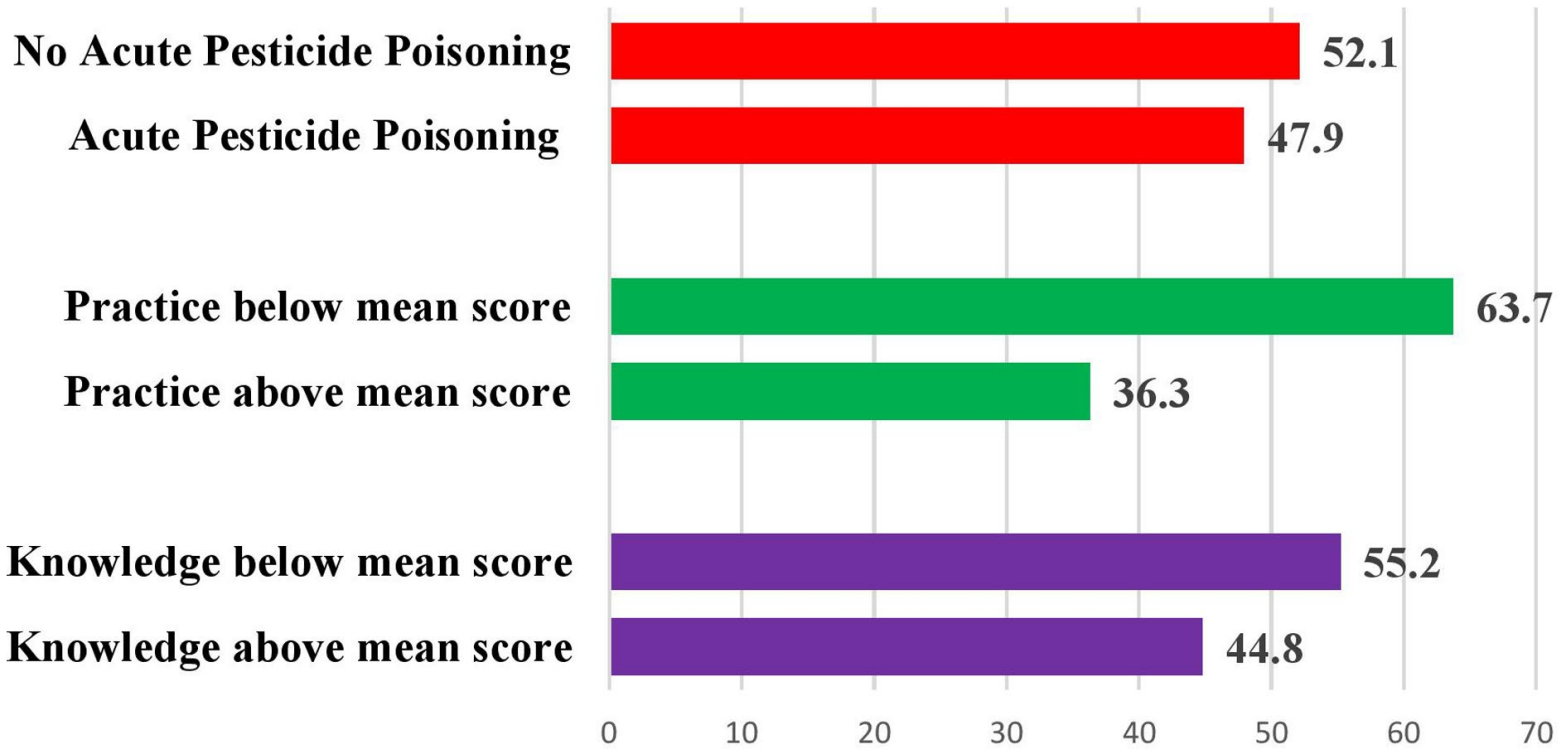
The overall magnitude of knowledge, safety practice, and acute pesticide poisoning.

Based on this study findings, 275 (44.8%) with 95% CI: 40.9–48.7, 223 (36.3%) 95% CI: 32.6–39.8, and 294 (47.9%) with 95% CI: 44.2–52.3 of farmers had above mean knowledge of health risk, above mean safety practice, and APP associated with use of pesticides, respectively, (Figure 2).

### Factors associated with knowledge of health risk for pesticide use

The multivariable logistic regression analysis revealed that farmers with no formal education (AOR = 2.32; 95% CI: 1.27–4.23) and those lacking professional training on pesticide use (AOR = 2.07; 95% CI: 1.33–3.21) were significantly associated with below mean knowledge of health risks (Table 6).

**Table 6:** Bivariable and multivariable logistic regression analysis of factors associated with knowledge of health risk among farmers in rural irrigation areas of South Wollo zone, Ethiopia, July–August, 2022.

Variable	Category	Knowledge		COR(95%CI)	AOR(95%CI)
		Below mean	Above mean		
Household type	Non model	113	175	1	1
	Model	162	164	1.53(1.11–2.11)	1.48(0.99–2.21)
Age of respondents	18–27	70	78	1	1
	28–37	112	144	0.86(0.58–1.30)	0.86(0.52–1.42)
	38–47	50	45	1.24(0.74–2.07)	1.37(0.73–2.56)
	>47	43	72	0.66(0.41–1.09)	0.79(0.43–1.44)
Educational status	No education	58	205	1	1
	Informal education	65	58	3.96(0.43–4.10)	3.78(2.34–6.11)
	Elementary education	51	38	4.74(2.45–6.47)	3.49(2.30–5.68)
	Secondary education	43	30	5.07(2.92–8.78)	6.02(3.35–10.81)
	College/University	27	39	2.45(1.38–4.33)	2.32(1.27–4.23)*
Years of exposure	3 years and below	129	97	1	1
	4–10 years	89	159	0.42(0.29–0.61)	0.54(0.34–0.85)
	>10 years	57	83	0.52(0.34–0.79)	0.60(0.36–1.01)
Training on pesticide use	No	183	267	1	1
	Yes	92	72	1.86(1.29–2.68)	2.07(1.33–3.21) *
Action of government	Poor	141	177	1	1
	Good	134	162	1.04(0.94–1.91)	1.84(1.22–2.79)

### Factors associated with safety practice during pesticide use

Multivariable logistic regression analysis output indicated that farmers age greater than 28 years old (AOR = 2.16; 95% CI: 1.17–3.97), below mean actions of pesticide retailers for farmers (AOR = 1.97; 95% CI: 1.34–2.89), and below mean attitude towards pesticide use (AOR = 1.79; 95% CI: 1.23–2.62) were significantly associated with below mean safety practice on pesticide use (Table 7).

**Table 7:** Bivariable and multivariable logistic regression analysis of factors associated with safety practice during pesticide use among farmers in rural irrigation areas of South Wollo zone, Ethiopia, July–August, 2022.

Variable	Category	Safety practice		COR(95%CI)	AOR(95%CI)
		Below mean	Above mean		
Household type	Non model	197	91	1	1
	Model	194	132	1.47(1.06–2.05)	1.04(0.69–1.56)
Age of respondents	18–27	93	55	1	1
	28–37	154	102	1.12(0.74–1.69)	2.16(1.17–3.97) *
	38–47	59	36	1.03(0.35–1.76)	2.09(1.19–3.67)
	>47	85	30	0.59(0.61–1.02)	2.07(1.06–4.04)
Educational status	No education	167	96	1	1
	Informal education	88	35	0.69(0.43–1.10)	1.19(0.63–2.28)
	Primary education	56	33	1.03(0.62–1.69)	0.62(0.30–1.28)
	Secondary education	37	36	1.69(1.03–2.86)	0.93(0.43–2.04)
	College/university	43	23	0.93(0.53–1.64)	1.73(0.79–3.77)
Wealth index	Lowest	72	133	0.73(0.91–2.04)	0.68(0.43–1.07)
	Medium	66	143	0.62(0.57–1.28)	0.73 (0.46–1.16)
	Highest	85	115	1	1
Type of crop grown	Cereals and legumes	117	59	1	1
	Fruits and vegetables	185	135	1.45(0.62–1.69)	0.67(0.36–1.25)
	Cash crops	89	29	0.65(0.38–1.09)	0.88 (0.50–1.54)
Years of exposure	3 years and below	125	101	2.25(1.42–3.56)	1.38(0.80–2.37)
	4–10 years	163	85	1.45(0.92–2.29)	1.18(0.69–1.99)
	>10 years	103	37	1	1
Pesticide training	No	302	148	1.72(1.19–2.48)	1.09(0.69–1.69)
	Yes	89	75	1	1
Action of retailors	Poor	181	137	0.54(0.39–0.76)	1.97(1.34–2.89) *
	Good	210	86	1	1
Trust in Retailors	Poor	128	58	1.38(0.96–1.99)	0.57(0.19–1.68)
	Good	263	165	1	1
Action of government	Poor	141	66	1.34(0.94–1.91)	1.05(0.37–2.98)
	Good	250	157	1	1
Knowledge	Poor	225	114	1.29(0.93–1.80)	1.17(0.75–1.83)
	Good	166	109	1	1
Attitude	Poor	196	136	0.64(0.46–0.89)	1.79(1.23–2.62) *
	Good	195	87	1	1

*Statistically significant at *p* < 0.05.

### Factors associated with self-reported acute pesticide poisoning

Multivariable logistic regression analysis output indicated that farmers with more than 10 years of exposure (AOR = 4.34; 95% CI: 2.55–7.39), below mean knowledge (AOR = 2.17; 95% CI: 1.38–3.40), lack of training about pesticide use (AOR = 3.73; 95% CI: 2.33–5.98), and below mean safety practice (AOR = 4.40; 95% CI: 2.86–6.78), were significantly associated with acute pesticide poisoning (Table 8).

**Table 8:** Bivariable and multivariable logistic regression analysis of factors associated with acute pesticide poisoning (APP) among farmers in rural irrigation areas of South Wollo zone, Ethiopia, July–August, 2022.

Variable	Category	APP		COR(95%CI)	AOR(95%CI)
		Yes	No		
Household type	Non model	183	105	3.38(2.42–4.70)	3.63 (2.43–5.41)
	Model	111	215	1	1
Marital status	Married	152	147	1	1
	Unmarried	142	173	0.79(0.58–1.09)	0.72 (0.49–1.07)
Educational status	No education	143	120	1.12(0.65–1.93)	1.02 (0.53–1.99)
	Informal education	56	67	0.78(0.43–1.43)	0.79(0.38–1.64)
	Elementary education	33	56	0.55(0.29–1.06)	1.14(0.50–2.56)
	Secondary education	28	45	0.59(0.29–1.15)	0.58(0.26–1.33)
	College/university	34	32	1	1
Years of exposure	3 years and below	63	163	1	1
	4–10 years	144	104	3.58(2.44–5.26)	3.45 (2.17–5.50)
	>10 years	87	53	4.25(2.71–6.65)	4.34 (2.55–7.39) *
Pesticide training	No	252	198	3.69(2.48–5.49)	3.73(2.33–5.98) *
	Yes	42	122	1	1
Knowledge of health risk	Poor	201	138	2.85(2.04–3.97)	2.17 (1.38–3.40) *
	Good	93	182	1	1
Attitude	Poor	167	165	1.23(0.89–1.69)	1.65 (1.10–2.46)
	Good	127	155	1	1
Safety practice	Poor	236	155	4.33(3.02–6.23)	4.40 (2.86–6.78) *
	Good	58	165	1	1

*Statistically significant at p < 0.05.

## Discussion

In this study, we measured the magnitude of knowledge of health risk, safety precaution practices, and self-reported acute pesticide poisoning and its predictors among farmers in rural irrigation areas of South Wollo Zone, Northeastern Ethiopia. Farmers in the study area had unsatisfactory knowledge of health risks, attitudes, and safety practices, as well as health issues related to pesticide use.

below mean below mean According to the findings of this study, more than half of the farmers scored below the mean in knowledge about the health risks of pesticides, and nearly two-thirds demonstrated safety practices that also were scored below the mean. About half of farmers consistently reported that they had at least two or more symptoms within 48 h of pesticide use in the previous 12 months. Contrary to previous findings, less than half of the farmers in this study were aware of the routes of pesticide exposure, which include ingestion and inhalation (15, 27). But far more than one-third of farmers knew that skin exposure was one way to be exposed to pesticide toxicity, compared to three-quarters of farmers who knew about the dermal exposure route from a Tanzanian study (27).

This study report states that 355 farmers (57.8%), 312 farmers (50.8%), and 236 farmers (38.4%) were aware that using pesticides could have long-term health effects, short-term health effects, and accumulate in the soil, respectively. These findings are lower than the findings of a study conducted in Kuwait, where farm workers reported that pesticides were harmful to both the environment (65%) and their own health (71%) (39).

In contrast to the study results from Gondar city, Ethiopia (39.4%) (17) and Central Eastern Ethiopian horticulture farms (31%) (40), this study found that 44.8% of farmers had above mean knowledge of health risk. These discrepancies may be attributed to better access to education and training programs, more effective agricultural extension services, and possibly more robust community outreach efforts in our study setting. However, the magnityde of above mean knowledge of health risk in this study was lower than findings from Southwest Ethiopia (54.4%) (6), from Tanzania (50%) (41), Lebanon (69.9%) (42), and from Gaza Strip (97.9%) (43). This disparity might be attributed to differences in educational initiatives, access to information, quality of training programs, and effectiveness of health communication strategies across study regions.

The magnitude of above mean safety practice in this study (36.3%) was higher than the study findings indicated from Central-Eastern Ethiopia (32%) (10). However, this study finding was lower than a study result reported from Butajira South Ethiopia (44.5%) (32), Southwest Ethiopia (58%) (6) and Kuwait (39). The difference in safety practices might be due to differences in educational programs, regulatory enforcement, and support from agricultural extension services. Cultural attitudes, the availability of protective equipment, and study methodologies also contribute to the variations observed.

In this study, the prevalence of self-reported acute pesticide poisoning was (47.9%) higher than study conducted in Central Eastern Ethiopia (32%) (10), in South India (33%) (30), in Northeastern Italy (43.5%) (21) and lower than in Central Rift valley, Ethiopia (55%) (40), in Tanzania (93%) (27), in Qianyang county, China (65%) (44), in Gaza Strip (78%) (43) and Kuwait (82%) (39). The disparity might be due to variations in reporting practices, pesticide use patterns, and agricultural techniques that influence prevalence rates. Differences in regulatory environments and healthcare access also play a role, as stricter regulations and better healthcare can lead to lower reported rates. Cultural attitudes towards pesticide use and safety practices, along with differences in study methodologies, contribute to the variations observed across study settings.

The present stsudy indicated that a number of factors, including the farmers’ educational background and training in pesticide use had a significant impact on their knowledge of health risks. Farmers who had higher levels of education perceived high risks (15) and above mean knowledge was obtained in China (44) and Italy (21) as result of training of farmers and effective involvement of the government.

Farmers’ below mean safety practice on pesticide use was due to increased age of farmers, lack of involvement of retailers on pesticide use, and below mean attitude. Older farmers are nearly twice as likely to have below mean safety practices compared to younger ones. This is due to reliance on outdated methods, reluctance to adopt new practices, physical limitations, and underestimating risks due to prolonged exposure. Younger farmers are more likely to have recent training and adapt to modern safety standards.

Farmers without the active involvement of retailers during pesticide purchases were nearly twice more likely to have below mean practices compared to active engagment of retailers. This finding is supported by a study conducted in rural China (45), Northern China (16) and Bangladesh (14). This might be due to the fact that retailers provide crucial information on the correct type, amount, and usage instructions for pesticides, which is essential for safe and effective application. They also advise on the necessity and proper use of personal protective equipment and educate farmers on safe handling and storage practices. They are also more aware of regulatory requirements and can help farmers comply with these rules, preventing misuse and potential penalties. Overall, retailer involvement bridges knowledge gaps, promotes safety, and ensures effective pesticide use among farmers.

The odds of having below mean safety practice among farmers with below mean attitude was doubled compared to those who have a above mean attitude and similar evidence were reported from Gondar city, Ethiopia (17) and Bangladesh (14). This can be justified by the fact that a negative attitude often correlates with a lack of motivation to follow safety protocols and adopt recommended practices. Farmers with below mean attitudes may be less inclined to seek out or value safety training, use protective equipment, or adhere to proper handling procedures. Moreover, they may underestimate the risks associated with pesticide use and be less responsive to educational efforts, leading to higher instances of unsafe practices.

The development of acute pesticide poisoning among farmers with varying exposure levels, knowledge, training, and safety practices is due to several factors. Specifically, the odds of developing APP due to over 10 years of pesticide exposure are more than four times compared to less than 3 years of exposure, more than twice as high due to below mean knowledge, nearly four times higher due to lack of pesticide training, and more than four times higher due to below mean safety practices compared to their counterparts. These findings are supported by evidence reported from Kermanshah, Iran (46), South India (30), Italy (21), and Turkey (47). Farmers with over 10 years of exposure accumulate more toxins, increasing their risk. Below mean knowledge and a lack of training result in unsafe handling practices and a lower perception of risk. Furthermore, inadequate safety practices, such as not using protective equipment, lead to higher direct exposure. Environmental factors, like local agricultural practices and pesticide types, also contribute to varying risks. Limited healthcare access and inconsistent quality of agricultural extension services further exacerbate the issue.

### Strength and limitations

This study holds practical importance in improving farmers’ knowledge of health risks and safety practices. It is crucial for reducing acute pesticide poisoning and maximizing productivity through practical implications for health and agriculture sector policymakers and program planners. The findings also significantly contribute to existing scientific evidence, demonstrating how farmers can apply and enhance their knowldege and safety practices.

However, the study is limited by its cross-sectional nature, which cannot guarantee a temporal relationship between causes and effect. Additionally, the snapshot nature of the study may not accurately reflect seasonal variations in safety practices and health effects of pesticide exposure. Therefore, to investigate the actual scenario, repeated seasonal-based studies may be required. Despite these limitations, this study used stanadardized and reliable tool with large sample size that enables to geneerate robust and truthworthy evidence on farmers’ knowledge of health risk, safety practices and pesticide-related acute poisoning in the study area.

## Conclusion

This study found that farmers’ awareness of health risks and safety practice was unsatisfactory, demonstrating that most farmers had inadequate awareness of health risks and subpar safety practice. Nearly half of the respondents experienced pesticide-related acute poisoning. These findings suggest that local health and agricultural departments should prioritize thorough health & safety education and oversight, particularly with regard to proper safety practices when using pesticides.

As adjusted multivariable logistic regression analysis indicated that low level of educational status, lack of training, and below mean involvement of government were significantly associated with below mean knowledge of health risk while being young farmers, lack of involvement of retailers on pesticide use, and below mean attitude were associated factors of below mean safety practice.

Long time exposure, below mean knowledge, below mean safety practice and lack of training were the main predictors of acute pesticide poisoning. These findings require urgent interventions from pesticides retailers, local government, and other stakeholders to reduce acute pesticide poisoning associated with pesticide use and protect the health of the farmers. To close the information gaps that farmers have regarding pesticides use, appropriate training programs on pesticide safety and the risks of pesticide exposure should be established. The national agricultural and health extension services ought to be at the forefront of providing farmers with up-to-date, reliable, and comprehensible information to encourage preventive practices.

## Data Availability

The original contributions presented in the study are included in the article/Supplementary material, further inquiries can be directed to the corresponding author.
